# Esophageal Schwannoma: An Important Differential Diagnosis for Esophageal Subepithelial Lesions

**DOI:** 10.7759/cureus.27168

**Published:** 2022-07-23

**Authors:** Bola Nashed, Mohamad F Ayas, Hussein Gharib, Mohamed Issa, Khairya Fatouh, Freny Sebastian, Zoheb Backer, Krishna Mahat, Mohammed Barawi

**Affiliations:** 1 Internal Medicine, Ascension St. John Hospital, Detroit, USA; 2 Pathology, Ascension St. John Hospital, Detroit, USA; 3 Gastroenterology, Ascension St. John Hospital, Detroit, USA

**Keywords:** endoscopic ultrasound (eus), submucosal tunneling, subepithelial lesions, esophagus, schwannoma

## Abstract

Esophageal schwannoma is a rare tumor and is classified as one of the esophageal subepithelial lesions (SELs). Endoscopic ultrasound (EUS) evaluation is the gold standard for evaluating subepithelial lesions. Differentiation through EUS-guided fine needle aspiration is sometimes important to exclude lesions with malignant potential. Immunohistochemistry differentiates schwannoma from other subepithelial lesions. Strong and diffuse positivity for S100 is characteristic. The decision for conservative management versus endoscopic or thoracoscopic intervention is made based on the tumor size, location, and symptoms.

## Introduction

Schwannomas are slow-growing, mostly benign tumors that arise from the Schwann cells of the nerve sheath [1]. They occur rarely in the gastrointestinal (GI) tract and represent about 2-6% of mesenchymal tumors arising in the GI tract [2]. They represent an important differential diagnosis for subepithelial lesions of the GI tract. The stomach is the most common site of presentation constituting around 70% of all gastrointestinal tract schwannomas. Meanwhile, esophageal schwannomas are rarely reported [1]. Here, we present a patient who presented with dysphagia due to an esophageal subepithelial mass that was found to be a schwannoma. 

## Case presentation

A 72-year-old female presented to our gastroenterology clinic for a complaint of dysphagia to solid food that had been progressing over a few months. She stated these symptoms started after her first COVID-19 vaccination around a year ago. 

We obtained an esophagogastroduodenoscopy (EGD) that showed a bulging lesion in the esophageal wall around 30 cm from the incisors, causing some luminal narrowing. We evaluated the mass using endoscopic ultrasound (EUS) and it revealed a subepithelial hypoechoic heterogeneous mass originating at the third or fourth layers of the esophagus. 

EUS-guided fine-needle aspiration biopsy (FNAB) was obtained, showing bland spindle cell proliferation with no atypia or mitosis. Cells showed S100 positivity, negative desmin, and smooth muscle actin (SMA), discovered on gastrointestinal stromal tumors 1 (GIST 1 (DOG-1)) and CD117 which suggests schwannoma. 

We obtained a chest MRI with contrast to further evaluate the mass. It confirmed a well-circumscribed 2.2 x 2.8 x 3.2 cm ovoid T2-hyperintense enhancing mass in the mid to distal esophagus that appeared to be submucosal.

**Figure 1:**
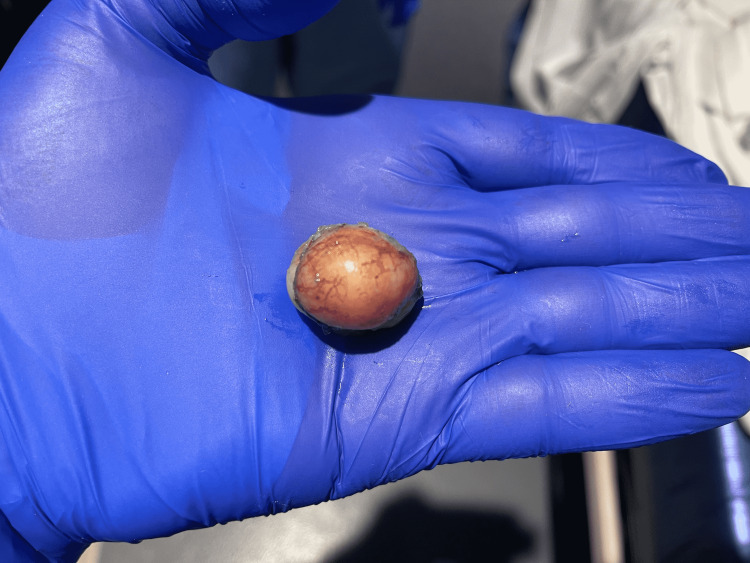
Tan-white polypoid soft tissue mass measuring 2.9 x 2.9 x 2.1 cm.

The patient continued to have dysphagia. After a discussion with the patient regarding her options, she elected to proceed with another EGD for resecting the mass using the submucosal tunnel endoscopic resection (STER) technique. A 10 mm mucosal incision was made 5 cm proximal to the mass. Dissection of the submucosal tissues was done and a tunnel was created. We dissected the mass from surrounding tissue till it was released from its attachments. The mass was captured and delivered and multiple endo clips were used to close the mucosal incision (Figure 1).

**Figure 2:**
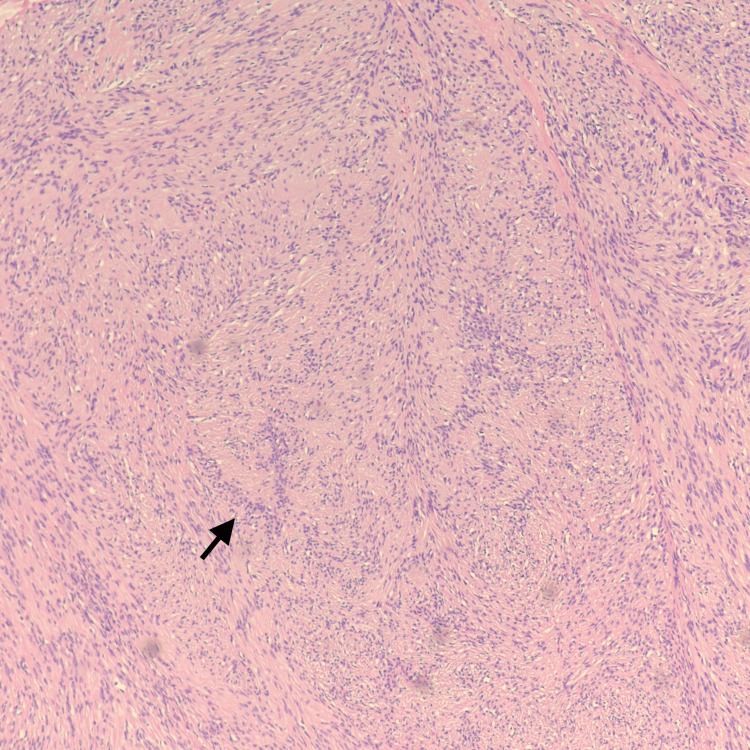
Characteristic microscopy findings of Antoni A area of schwannoma with Verocay bodies with nuclear palisading. (Hematoxylin and eosin, 100X)

**Figure 3:**
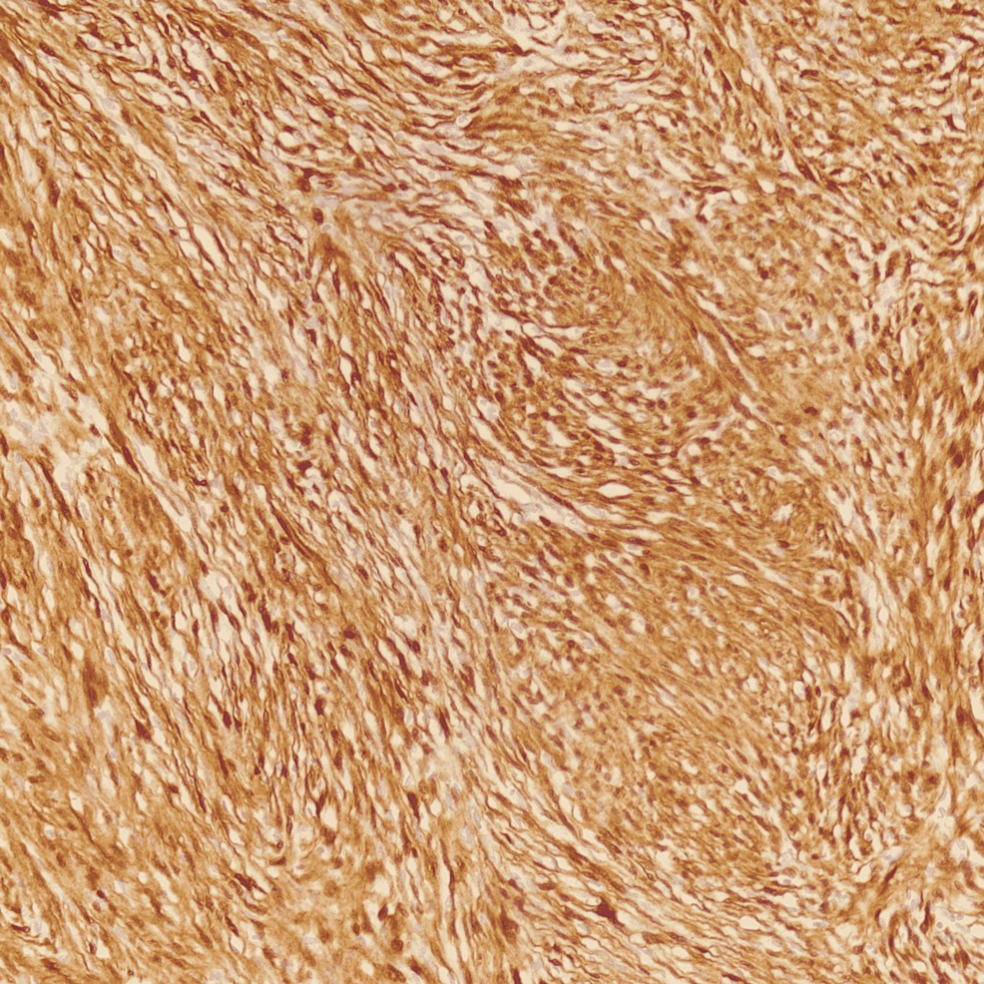
Immunohistochemical staining positive for S100 (200X)

Pathologic analysis showed a well-circumscribed lesion with alternating hypercellular Antoni A with nuclear palisading around fibrillary processes (Verocay bodies) and hypocellular areas (Figure 2). Immunohistochemical staining showed the same profile as the FNAB (Figure 3). Histologic and immunohistochemical profiling supporting the diagnosis of schwannoma. 

We obtained an esophagogram one day after the resection which was negative for any leakage; a liquid diet was introduced on Day 1 after the procedure then advanced to a soft diet, and the patient was discharged home after resolution of dysphagia. A follow-up EGD was done after two months, with a normal appearance of mucosa and improvement of the patient’s symptoms.

## Discussion

Subepithelial lesions of the gastrointestinal tract are increasingly discovered with the advancement in endoscopy. They are mostly benign at the time of diagnosis, however many of these lesions have malignant potential [3].

Endoscopic ultrasound (EUS) is of great value and is currently recommended as a gold standard to describe subepithelial lesions including size, location, originating layer, echogenicity, and shape however, it's not enough to absolutely identify the lesion [4]. Currently, the European Society of Gastrointestinal Endoscopy recommends tissue diagnosis if the subepithelial lesion (SEL) has features suggestive of GIST, if they are of size > 20 mm, have high-risk stigmata, or if they require surgical resection or oncological treatment [4]. 

Esophageal schwannoma is very rare and few cases are reported in the literature. The most common presenting symptom is dysphagia. On EUS, the schwannoma has sharp borders, hypoechoic, homogeneous, and sometimes with a marginal halo [4]. In our patient, the mass appeared as a subepithelial hypoechoic heterogeneous mass originating at the third or fourth layers of the esophagus. 

Diagnosis is through tissue biopsy using immunohistochemical staining. Positivity for S100 protein and negativity for actin and desmin, support a nerve sheath origin of the tumor [3]. In our patient, tissue biopsy showed S100 positivity, negative desmin, smooth muscle actin (SMA), discovered on GIST 1 (DOG-1) and CD117. The negative staining for desmin and SMA made leiomyoma less likely and the negative staining for CD117 and DOG1 made gastric stromal tumor less likely. Positive staining for S100 favored schwannoma diagnosis. 

Management is usually based on the presence or absence of symptoms. If obstructive symptoms are present, endoscopic full-thickness resection should be compared with thoracoscopic enucleation. The upper limit for the endoscopic approach is 35 mm to allow en bloc removal. Submucosal tunneling endoscopic approach is recommended [4]. In our patient, resection was decided as the patient was symptomatic with dysphagia. 

## Conclusions

Esophageal schwannoma is a rare gastrointestinal tract tumor. It is an important differential diagnosis of subepithelial tumors of the gastrointestinal tract. There is not enough evidence in the literature to suggest that EUS tissue acquisition is required from all SELs or only from those > 20 mm or with high-risk stigmata. However, multiple gastroenterology societies recommend obtaining tissue biopsy for SELs with a size > 20 mm, or have high-risk stigmata since definite differentiation from other SELs is not feasible through EUS characterization alone. 
